# Supplementary material for: Content-rich biological network constructed by mining PubMed abstracts
Source: BMC Bioinformatics. 2004 Oct 8;5:147. doi: 10.1186/1471-2105-5-147 (PMC528731; doi:10.1186/1471-2105-5-147)
Supplement: Additional File 2 — The original results of the above study (non-essential files are deleted to keep the file size under the limit set by BMC bioinformatics). [file 1471-2105-5-147-S2.bz2 › chilibotAdditionalFile2/dip10/763ID9732274E18151/html/ENO_RNE.html]

 


 **ENO** and **RNE** 
  
Found 10 abstracts in PubMed, retrieved 10.  
 

 What does Google say? 
 PDF only 
| .edu only 

---

**Interactive relationship** (e.g. stimulation, inhibition, etc)

**Neutral relationship**- Earlier work has shown that the C terminal region of RNase E  [ **RNE** ]  is a scaffold for the binding of degradosome components and has identified specific RNase E  [ **RNE** ]  segments necessary for its interaction with polynucleotide phosphorylase PNPase, RhlB RNA helicase, and enolase  [ **ENO** ] .  Ref: 11134527 Proc Natl Acad Sci U S A, 2001
- Our results demonstrate that the carboxy terminal half CTH of ribonuclease E  [ **RNE** ]  RNase E  [ **RNE** ]  contains the binding sites for the three other major degradosomal components, the DEAD box RNA helicase RhlB, enolase  [ **ENO** ] , and polynucleotide phosphorylase PNPase.  Ref: 9732274 Genes Dev, 1998
- RNase E  [ **RNE** ]  is a large multidomain protein with N terminal ribonucleolytic activity, an RNA binding domain and a C terminal scaffold that binds PNPase, enolase  [ **ENO** ]  and RhlB.  Ref: 12035760 Biochem Soc Trans, 2002
- We also show that the C terminal half of E. coli RNase E  [ **RNE** ]  is both sufficient and necessary for its physical interaction with the 3 5 exoribonuclease polynucleotide phosphorylase, the RhlB helicase, and the glycolytic enzyme enolase  [ **ENO** ] , which are components of a degradosome complex.  Ref: 9751718 Proc Natl Acad Sci U S A, 1998

**Non-interactive relationship** (e.g. studied together, co-existance, homology, etc.)

- The Escherichia coli degradosome is a multienzyme complex with four major protein components the endoribonuclease RNase E  [ **RNE** ] , the exoribonuclease PNPase, the RNA helicase RhlB and enolase  [ **ENO** ] .  Ref: 9383162 Mol Microbiol, 1997
- N terminal sequencing of these proteins revealed the presence in the RNase E  [ **RNE** ]  complex not only of polynucleotide phosphorylase but also of DnaK, RNA helicase, and enolase  [ **ENO** ] .  Ref: 8632981 Proc Natl Acad Sci U S A, 1996
- Using an E. coli two hybrid system, together with BIAcore apparatus, we investigated the ability of three proteins, polynucleotide phosphorylase PNPase, RhlB RNA helicase, and enolase  [ **ENO** ] , a glycolytic protein, to interact physically and functionally independently of RNase E  [ **RNE** ] .  Ref: 12181321 J Biol Chem, 2002
- Whereas PNPase and enolase  [ **ENO** ]  are present in E. coli in large excess relative to RNase E  [ **RNE** ]  and therefore are detected in cells largely as molecules unlinked to the RNase E  [ **RNE** ]  scaffold, immunogold labeling and biochemical analyses show that helicase is present in approximately equimolar amounts to RNase E  [ **RNE** ]  at all cell growth stages.  Ref: 11134527 Proc Natl Acad Sci U S A, 2001
- In Escherichia coli, the exoribonuclease polynucleotide phosphorylase PNPase, the endoribonuclease RNase E  [ **RNE** ] , a DEAD RNA helicase and the glycolytic enzyme enolase  [ **ENO** ]  are associated with a high molecular weight complex, the degradosome.  Ref: 11680851 RNA, 2001
